# Genome-Wide Identification and Expression Pattern Analysis of the Late Embryogenesis Abundant (LEA) Family in Foxtail Millet (*Setaria italica* L.)

**DOI:** 10.3390/genes16080932

**Published:** 2025-08-04

**Authors:** Yingying Qin, Yiru Zhao, Xiaoyu Li, Ruifu Wang, Shuo Chang, Yu Zhang, Xuemei Ren, Hongying Li

**Affiliations:** 1 College of Life Sciences, Shanxi Agricultural University, Jinzhong 030800, China; 15726077264@163.com (X.L.); 18786533470@163.com (R.W.); 17503368138@163.com (S.C.); 13834999791@163.com (Y.Z.); renxuemei@sxau.edu.cn (X.R.); 2 Houji Laboratory in Shanxi Province, Taiyuan 030031, China; zyr1533446379@163.com; 3 College of Agriculture, Shanxi Agricultural University, Jinzhong 030800, China

**Keywords:** foxtail millet, *LEA* gene family, genome-wide identification, bioinformatic analysis, ABA response, expression analysis

## Abstract

**Background/Objectives**: Late embryogenesis abundant (LEA) proteins regulate stress responses and contribute significantly to plant stress tolerance. As a model species for stress resistance studies, foxtail millet (*Setaria italica*) lacks comprehensive characterization of its *LEA* gene family. This study aimed to comprehensively identify *SiLEA* genes in foxtail millet and elucidate their functional roles and tissue-specific expression patterns. **Methods**: Genome-wide identification of *SiLEA* genes was conducted, followed by phylogenetic reconstruction, *cis*-acting element analysis of promoters, synteny analysis, and expression profiling. **Results**: Ninety-four *SiLEA* genes were identified and classified into nine structurally distinct subfamilies, which are unevenly distributed across all nine chromosomes. Phylogenetic analysis showed closer clustering of *SiLEA* genes with sorghum and rice orthologs than with *Arabidopsis thaliana AtLEA* genes. Synteny analysis indicated the *LEA* gene family expansion through tandem and segmental duplication. Promoter *cis*-element analysis linked *SiLEA* genes to plant growth regulation, stress responses, and hormone signaling. Transcriptome analysis revealed tissue-specific expression patterns among *SiLEA* members, while RT-qPCR verified ABA-induced transcriptional regulation of *SiLEA* genes. **Conclusions**: This study identified 94 *SiLEA* genes grouped into nine subfamilies with distinct spatial expression profiles. ABA treatment notably upregulated *SiASR-2*, *SiASR-5*, and *SiASR-6* in both shoots and roots.

## 1. Introduction

Plants activate specific response programs under abiotic stress, enhancing stress resistance through the production of protective proteins that preserve cellular homeostasis [1,2]. Among these, late embryogenesis abundant (LEA) proteins show marked stress-inducible expression, making them prominent subjects in plant stress research [3]. Although their roles have been thoroughly characterized in model plants such as *Arabidopsis* [4], sorghum (*Sorghum bicolor*) [5], rice (*Oryza sativa*) [6], and wheat (*Triticum aestivum*) [7], LEA proteins also occur in fungi, bacteria, and metazoans [8]. Unlike typical proteins that follow the “sequence-structure-function” paradigm, LEA proteins often lack stable tertiary structures and belong to the intrinsically disordered protein class [9].

The *LEA* family includes numerous members, and its taxonomic classification has been revised several times. Early nomenclature assigned *LEA* family members with a capital “D” followed by numerals, such as D-19 and D-29 [10]. Based on conserved motif divergence in LEA protein sequences, these proteins are classified into six subfamilies [11]. After the establishment of the Late Embryogenesis Abundant Proteins Database (LEAPdb), the classification expanded to eight subfamilies according to PFAM conserved domain architecture [12], namely *LEA*_1 (PF03760), *LEA*_2 (PF03168), *LEA*_3 (PF03242), *LEA*_4 (PF02987), *LEA*_5 (PF00477), *LEA*_6/*PvLEA*18 (PF10714), *Dehydrin* (PF00257), and *SMP* (Seed maturation protein, PF04927). ASR (abscisic acid, stress, and ripening-induced) proteins constitute a distinct subgroup of plant LEA proteins. These genes show coordinated expression with canonical *LEA* genes and encode intrinsically disordered proteins with physicochemical similarities to LEA proteins. Thus, ASR proteins are classified as the *LEA*_7 subfamily, extending the *LEA* classification to nine subfamilies [13]. Two additional proteins without PFAM domain classification were identified as members of the *AtM* subgroup in the *Arabidopsis LEA* gene family [3].

*LEA* family members play crucial roles in plant adaptation to abiotic stresses. In *Arabidopsis*, numerous *AtLEA* genes show strong induction under stress conditions. Mutational studies reveal that *Atlea4* loss-of-function mutants exhibit increased drought sensitivity [14], whereas *AtLEA5* regulates antioxidant defense systems [15]. Salt stress markedly upregulates *AtLEA14* expression, and both transgenic *Arabidopsis* and heterologous yeast systems overexpressing this gene demonstrate improved salinity tolerance [16]. *AtLEA4-5* transcription increases under salt or osmotic stress, confirming its role in drought tolerance mechanisms [17]. Transgenic rice and wheat expressing barley (*Hordeum vulgare*) *HVA1* (*LEA*_3 subfamily) show improved water-use efficiency under drought conditions [18,19]. Similarly, rice overexpressing *OsEm1* (*LEA*_1) or *OsLEA5* (*LEA*_2) exhibits enhanced drought and salt tolerance [20,21]. Drought stress substantially elevates *ZmLEA34* expression in maize (*Zea mays*), while *ZmNHL1* overexpression improves drought resistance by enhancing ROS scavenging and maintaining membrane integrity [22,23]. Foxtail millet *SiLEA14* responds to osmotic stress, NaCl, and exogenous ABA; its heterologous expression in *Arabidopsis* improves seedling stress tolerance, and overexpressing foxtail millet lines show better physiological performance under salt and drought conditions [24]. In cotton (*Gossypium hirsutum*), *GhLEA-5A/D* may positively regulate salt stress responses [25]. *GhLEA3* knockout increases sensitivity to salinity and drought while reducing ABA/stress-responsive gene expression, whereas overexpression enhances stress tolerance [26]. Tobacco (*Nicotiana tabacum*) expressing kenaf (*Hibiscus cannabinus*) *HcLEA113* gains drought resilience, while its silencing impairs growth under drought [27]. Pepper (*Capsicum annuum*) *CaLEA1* modulates drought and salt tolerance via ABA signaling, with overexpressing lines showing ABA-hypersensitive germination, improved stomatal regulation, and upregulated stress genes [28]. *Medicago sativa* with *MsLEA1* overexpression gains drought and aluminum tolerance, while silencing reduces stress resilience [29]. Soybean (*Glycine max*) *GmLEA4_19* expression in *Arabidopsis* increases plant height under drought, and transgenic soybean lines show enhanced drought tolerance [30]. *Brassica campestris BcLEA73* improves root elongation, germination under stress, and drought survival in *Arabidopsis* [31]. *Rosa chinensis RcLEA* enhances growth recovery after temperature extremes [32]. *Medicago truncatula MtPM25* prevents stress-induced protein aggregation and dissolves existing aggregates via chaperone activity [33]. *MsLEA69* from *M. sativa* increases osmotic and temperature tolerance in prokaryotic and eukaryotic systems by elevating stress enzyme activity [34]. *Brassica napus* with *BnaA.LEA6.a* overexpression shows greater freezing resistance [35], while *Pinus tabuliformis PtLEA22* improves freezing tolerance when expressed in *Arabidopsis* [36].

Foxtail millet (*S. italica*), an annual C_4_ herbaceous plant in the Poaceae family, is both a traditional Chinese crop [37] and a promising model organism owing to its drought tolerance, adaptability to poor soils, storage stability, compact genome, and experimental convenience [38,39]. However, the *LEA* gene family in foxtail millet has not been thoroughly studied. Here, we identify all *SiLEA* family members and analyze their chromosomal locations, phylogenetic relationships, conserved domains, gene structures, promoter *cis*-elements, collinearity patterns, and expression profiles. We also examine the ABA-responsive expression patterns of *SiLEA* genes, providing foundational insights into their functional roles in this species.

## 2. Materials and Methods

### 2.1. Identification and Chromosomal Localization Analysis of SiLEA Gene Family Members in Foxtail Millet

Foxtail millet genomic resources—whole-genome sequences, CDS annotations, .gff structural annotation files, and complete proteome amino acid sequences—were obtained from Multi-omics Database for *S. italica* (MDSi, http://foxtail-millet.biocloud.net, accessed on 20 October 2023) [40]. The Hidden Markov Model (HMM) profile for LEA protein domains was acquired from the Pfam database (http://pfam-legacy.xfam.org/, accessed on 27 October 2023). We identified candidate *SiLEA* family genes by performing an HMMER scan (E-value ≤ 1 × 10^−5^) against the *S. italica* reference proteome (v2.2) (https://www.ebi.ac.uk/interpro/entry/pfam, accessed on 27 October 2023). The amino acid sequences from rice [6], *Arabidopsis * [3], and sorghum [5] were aligned with their foxtail millet counterparts using TBtools(v2.322)’ BLAST function [41], with candidate genes for the *SiLEA* family selected based on an E-value threshold of <0.001. After identifying *SiLEA* gene family members through Venn diagram analysis of gene counts obtained by both methods, we mapped their chromosomal locations using the foxtail millet .gff annotation file and visualized the results with TBtools (v2.322)’ Graphics function [41].

### 2.2. Physicochemical Properties of SiLEA Gene Family Members in Foxtail Millet

The amino acid composition and physicochemical properties of the *SiLEA* gene family were characterized using ExPaSy ProtParam (http://web.expasy.org/protparam, accessed on 21 March 2024). Subcellular localization predictions were performed with PSORT (http://psort1.hgc.jp/form.html, accessed on 10 April 2024).

### 2.3. Phylogenetic Analysis of Foxtail Millet SiLEA Gene Family Members

We aligned LEA amino acid sequences from foxtail millet, rice [6], *Arabidopsis* [3], and sorghum [5] using ClustalW and constructed a phylogenetic tree with the Neighbor-Joining (NJ) approach in MEGA 11.0 [42]. The phylogenetic tree was further annotated and visualized using the iTOL online tool (https://itol.embl.de/, accessed on 10 April 2024).

### 2.4. Conserved Domain and Gene Structure Analysis of Foxtail Millet SiLEA Genes

The NCBI-CDD Batch CD-Search tool (https://www.ncbi.nlm.nih.gov/Structure/bwrpsb/bwrpsb.cgi, accessed on 18 April 2024) identified conserved domains in the SiLEA protein sequences, which were then visualized with TBtools (v2.322) software [41]. Gene structures were reconstructed using the TBtools (v2.322) Gene Structure View (Advanced) tool [41], incorporating the foxtail millet genome annotation (.gff) and corresponding gene IDs.

### 2.5. Analysis of SiLEA Gene Promoter Cis-Regulatory Elements in Foxtail Millet

We extracted the promoter sequences of *SiLEA* genes using TBtools (v2.322) [41]. The PlantCARE database (http://bioinformatics.psb.ugent.be/webtools/plantcare/html/, accessed on 26 April 2024) identified *cis*-regulatory elements, which were then visualized with TBtools (v2.322) [41].

### 2.6. Collinearity and Evolutionary Patterns of SiLEA Genes

Interspecies collinearity of *LEA* genes and intraspecies collinearity of *SiLEA* genes were analyzed using genome annotations and sequences from foxtail millet, rice [6], sorghum [5], and *Arabidopsis* [3] through TBtools (v2.322)’ One Step MCScanX-SuperFast tool [41]. Collinear gene pairs were also visualized using TBtools (v2.322) [41]. For foxtail millet segmental duplications, Ka, Ks, and Ka/Ks ratios were calculated with TBtools (v2.322)’ Simple Ka/Ks Calculator [41].

### 2.7. Tissue-Specific Expression Analysis of Foxtail Millet SiLEA Gene Family Members

Transcriptome data for *SiLEA* gene family members across multiple tissues and developmental stages were obtained from the MDSi [40]. The dataset comprised 3-day germinated seeds, seedlings at the two-leaf and one-heart stage, heading-stage leaves, roots/stems/leaf veins during grain filling, panicles at various developmental phases, and mature seeds. Expression heatmaps were generated using TBtools (v2.322) [41].

Root samples were collected from 9-day-old Yugu1 seedlings treated with 2 µM ABA and untreated controls (CK), grown in an artificial climate chamber under 30,000 lux light intensity with a 16 h photoperiod at 28 °C and an 8 h dark period at 22 °C. Jingu 21 young panicles were sampled at the branch meristem differentiation phase (young panicle stage 1, ~1.0–1.5 mm) and the distinct formation phase (young panicle stage 2, ~2.5–3.0 mm). All collected root and young panicle samples were immediately flash-frozen in liquid nitrogen and stored at −80 °C. Three biological replicates were analyzed per sample. Novogene Co., Ltd. (Beijing, China) performed RNA library preparation and subsequent high-throughput sequencing.

### 2.8. Expression Analysis of SiLEA Gene in Response to ABA in Different Tissues of Foxtail Millet by qRT-PCR

Foxtail millet seeds were rinsed with deionized water, surface-sterilized in 70% ethanol for 30 s, and then immersed in 5% NaClO for 20 min before a final rinse with deionized water. The sterilized seeds were evenly distributed on moist filter paper (three layers) in plate and incubated in darkness for 12 h before being transferred to a growth chamber under standard conditions. The 5-day-old seedlings showing uniform growth were transplanted into hydroponic boxes containing 2 μM ABA solution. Shoots and roots were collected at 0, 12, 24, and 48 h after ABA treatment, immediately flash-frozen in liquid nitrogen, and stored at −80 °C. Each treatment included three biological replicates.

Total RNA was extracted from −80 °C preserved root and leaf samples of ABA-treated seedlings. cDNA synthesis was performed using the UnionScript First-strand cDNA Synthesis Mix (with dsDNase) (Genesand Biotech Co., Ltd., Beijing, China) for Real-time Quantitative Polymerase Chain Reaction (RT-qPCR), and the resulting cDNA was stored at −20 °C. Gene-specific primers for *SiLEA* genes (Table S1) were designed to amplify fragments of 80–200 bp. RT-qPCR was conducted on a Bio-Rad CFX 96 Real-Time PCR Detection System, with *Si9g37480* serving as the internal control [38]. Three biological replicates, each with three technical replicates, were analyzed. Statistical significance was assessed using ANOVA (Analysis of Variance) in GraphPad Prism(v9.0.0), with results expressed as means ± SE.

## 3. Results

### 3.1. Identification, Physicochemical Characterization, and Chromosomal Localization of SiLEA Gene Family Members in Foxtail Millet

**Figure 1:**
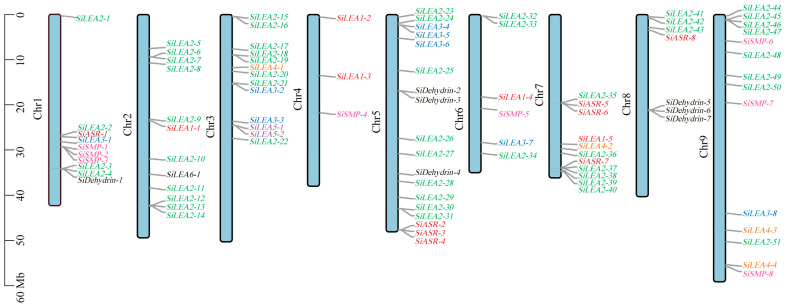
Chromosome localization map of *SiLEA* genes in foxtail millet. Genes from different subfamilies were assigned different colors for visual distinction.

Initial HMMER analysis identified 119 candidate *LEA* family genes, which were reduced to 98 after Blastp screening. PFAM domain verification confirmed 94 genes with authentic LEA domains (Table 1, Figure 1). Based on conserved domains and PFAM classifications, these genes clustered into nine subfamilies, *LEA*_1–*LEA*_6*/PvLEA18*, *ASR*, *Dehydrin* and *SMP* (Table 1, Figure 1). The *SiLEA*_2 subfamily was the largest, encompassing 51 genes designated as *SiLEA2-1* to *SiLEA2-51* (Table 1, Figure 1). The *SiLEA3, SiSMP*, and *SiASR* subfamilies each contained eight genes, designated as *SiLEA3-1* to *SiLEA3-8*, *SiSMP-1* to *SiSMP-8*, and *SiASR-1* to *SiASR-8*, respectively (Table 1, Figure 1). Seven genes formed the *SiDehydrin* subfamily (*SiDehydrin1* to *SiDehydrin7)*, while the *SiLEA_1* subfamily contained five (*SiLEA1-1* to *SiLEA1-5)* (Table 1, Figure 1). The *SiLEA*_4, *SiLEA*_5, and *SiLEA_6* subfamilies comprised four, two, and one gene (s), respectively (Table 1, Figure 1).

Characterization of the physicochemical properties of the *SiLEA* gene family (Table 1) showed that these genes encode proteins with lengths varying between 90 (*SiLEA3-1*) and 641 (*SiLEA2-40*) amino acids. The corresponding molecular masses span from 9.41 KDa (SiLEA3-1) to 70.62 KDa (SiLEA2-40). The isoelectric points (pI) of these proteins range from 4.63 (SiSMP-4) to 11.98 (SiLEA3-2), with most exhibiting alkaline pI values. Subcellular localization predictions suggest predominant targeting to chloroplast (36%), cytoplasm (22%), and nuclei (18%). The majority of SiLEA proteins demonstrate characteristic hydrophilicity (Table 1).

Chromosomal localization analysis (Table 1, Figure 1) showed an uneven distribution of *SiLEA* gene family members across the nine chromosomes of foxtail millet. Chromosome 5 contained the highest concentration with 18 genes (19.15% of total), whereas chromosome 4 had only three members (3.19%). The remaining chromosomes displayed intermediate counts: 10 genes on chromosome (chr) 1; 12 on chr 2; 13 on chr 3; 6 on chr 6; 11 on chr 7; 7 on chr 8; and 14 on chr 9 (Table 1, Figure 1).

### 3.2. Phylogenetic Analysis of SiLEA Gene Family Members

**Figure 2:**
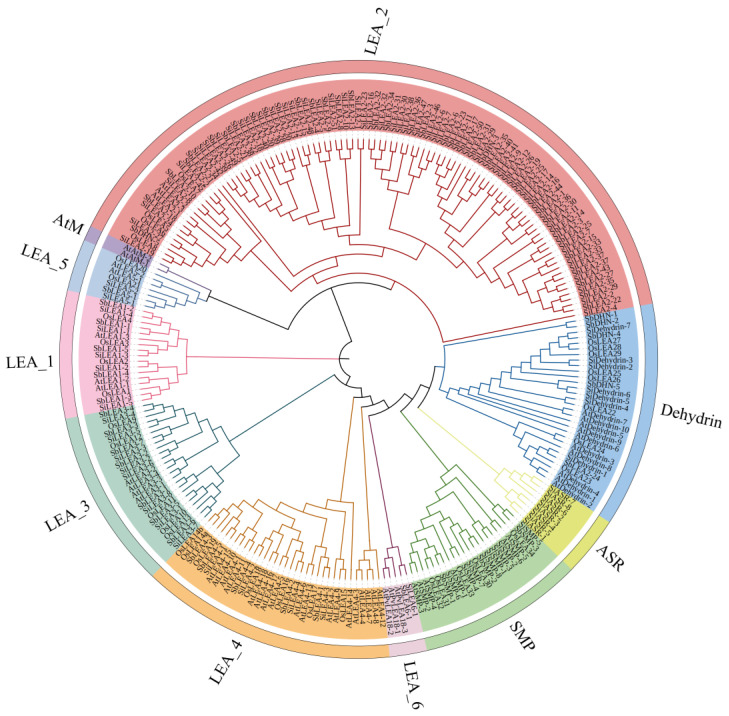
Phylogenetic analysis diagram of *LEA* genes in *S. italica* (*Si*), *O. sativa* (*Os*), *S. bicolor* (*Sb*), and *Arabidopsis* (*At*).

Phylogenetic relationships among *LEA* gene family members were reconstructed using amino acid sequences of 246 *LEA* genes—including 94 *SiLEA* genes from foxtail millet, 34 *OsLEA* genes from rice, 67 *SbLEA* genes from sorghum, and 51 *AtLEA* genes from *Arabidopsis*—were subjected to multiple sequence alignment and phylogenetic tree construction (Table S2). The analysis grouped these genes into 10 distinct subfamilies, *LEA*_1—*LEA*_6/*PvLEA*18, *ASR*, *Dehydrin*, *SMP*, and *AtM* subfamilies (Figure 2). These subfamilies further segregated into three major clades: Clade I (*LEA*_2, *LEA*_5, *AtM*); Clade II (*LEA*_1); Clade III (*LEA*_3, *LEA*_4, *LEA*_6/*PvLEA*18, *ASR*, *Dehydrin*, *SMP*). Species-specific expansion patterns emerged, with foxtail millet and sorghum showing pronounced gene duplication in the *LEA*_2, while *Arabidopsis* exhibited higher representation in the *LEA*_4 and Dehydrin. Although most subfamilies formed well-defined clusters (e.g., *LEA*_3/*LEA*_4), exceptions were noted. *SbDHN-3* and *SbDHN-5* from sorghum *Dehydrin* nested within *LEA*_2, and *SbLEA2-24* anomalously grouped with the *Dehydrin*, implying potential functional overlap or conserved structural features. The *AtM* and *ASR* subfamilies displayed lineage-specific divergence: *Arabidopsis AtM* genes (*AtAtM*, 2 members) formed an isolated branch, whereas foxtail millet *ASR* genes (*SiASR*, 8 members) clustered independently, indicating divergent functional specialization (Figure 2).

### 3.3. Conserved Domains and Gene Structures of SiLEA Genes in Foxtail Millet

**Figure 3:**
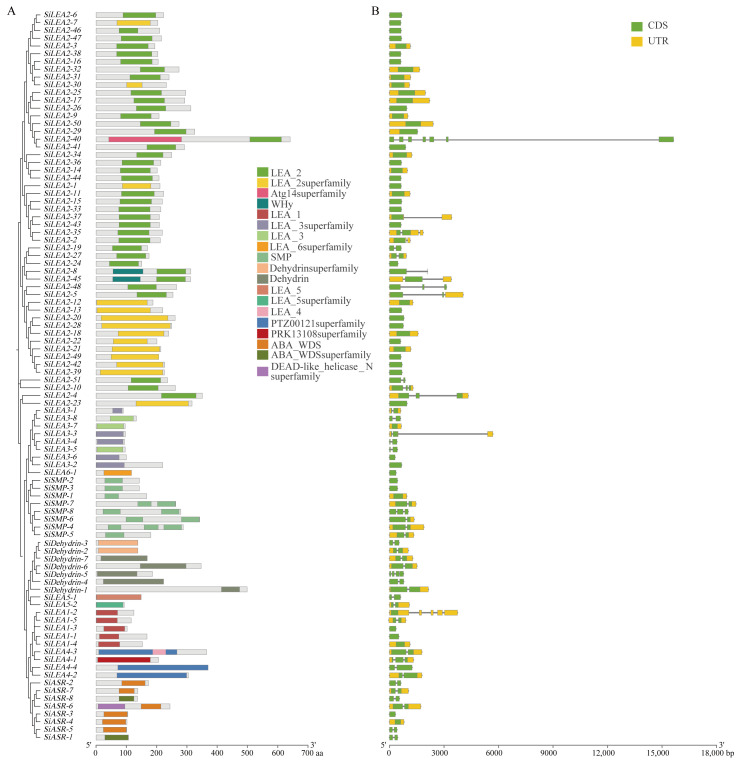
Conserved domains (**A**) and gene structures (**B**) of the *SiLEA* gene family in foxtail millet.

Conserved domain analysis revealed that *SiLEA* genes encode proteins with characteristic subfamily-specific domains: LEA_1 for *SiLEA*_1, LEA_3/LEA_3 superfamily for *SiLEA*_3, LEA_5/LEA_5 superfamily for *SiLEA*_5, LEA_6 superfamily for *SiLEA*_6, SMP for *SiSMP*, and Dehydrin/Dehydrin superfamily for *SiDehydrin* (Figure 3A). The *SiLEA*_2 subfamily proteins all contain the core LEA_2 domain, though some members display additional domains—SiLEA2-40 protein carries an N-terminal Atg14 superfamily domain, while SiLEA2-8 and SiLEA2-45 proteins feature N-terminal WHy domains. In the *SiLEA*_4 subfamily, SiLEA4-3 protein contains both LEA4 and PTZ00121 superfamily domains; whereas SiLEA4-2 and SiLEA4-4 proteins possess only the PTZ00121 superfamily domain; SiLEA4-1 protein uniquely encodes a PRK13108 superfamily domain. All *SiASR* subfamily proteins contain the ABA_WDS domain, with SiASR-6 protein additionally harboring an N-terminal DEAD-like_helicase_N superfamily domain, demonstrating strong sequence conservation across the gene family (Figure 3A).

Further analysis of gene structures demonstrated that most *SiLEA* genes are split, with 46.81% being intronless (Figure 3B). Forty-nine genes completely lack both 5′- and 3′-UTRs, while 43 retain both UTR regions; exceptions include *SiLEA2-29* (5’-UTR only) and *SiSMP-6* (3’-UTR only). The number of exons ranges from 1 to 7, indicating considerable structural and functional divergence among *SiLEA* family members (Figure 3B).

### 3.4. Promoter Cis-Acting Elements in SiLEA Genes

**Figure 4:**
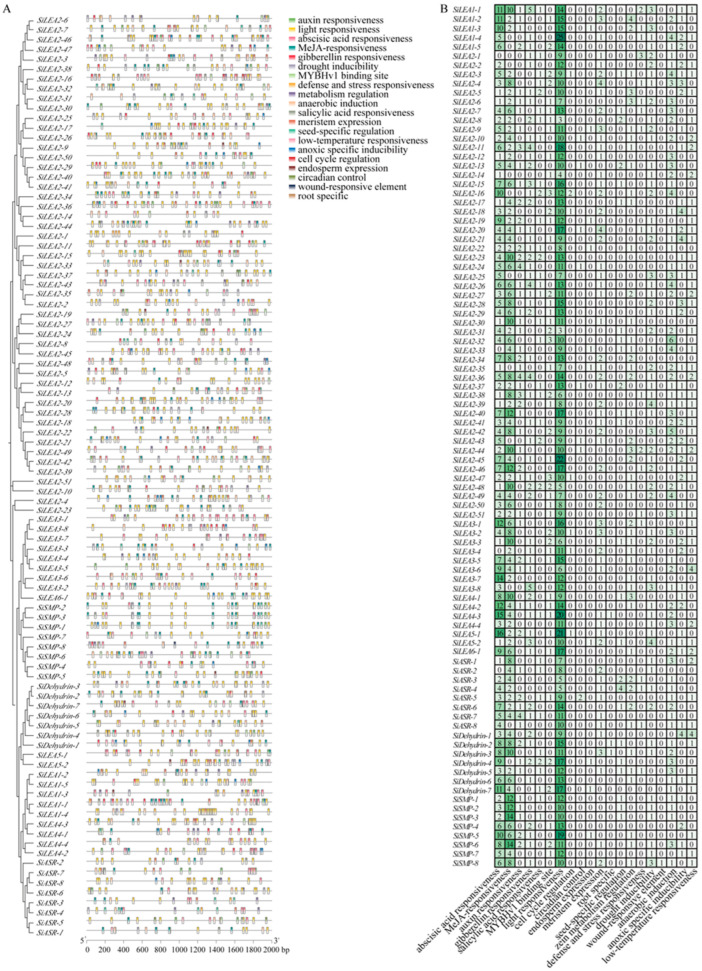
*Cis*-acting elements in promoter regions of *SiLEA* family genes in foxtail millet. (**A**) Distribution of *cis*-elements in promoter regions. Differently colored blocks represent distinct types of *cis*-elements and their positions within the 2000-bp upstream region of *SiLEA* genes. (**B**) *Cis*-element composition in promoters of individual *SiLEA* genes. Colors in the grid correspond to distinct *cis*-element types, and numbers indicate their counts, with vertical color bars corresponding to specific element types.

Analysis of *cis*-acting elements in *SiLEA* promoter regions revealed three functional categories (Figure 4, Table S3), providing insights into their potential roles in stress responses. Class I, associated growth and development elements, includes light responsiveness (present in all 94 *SiLEA* genes), root-specific elements, cell cycle regulation elements, alongside meristem expression, MYBHv1 binding sites, endosperm expression, and circadian control elements. Class II, linked to biotic and abiotic stress elements, feature drought inducibility, low-temperature responsiveness, wound-responsive elements, and defense/stress responsiveness, indicating these genes predominantly contribute to drought and cold adaptation. Class III elements mediate hormone response, with ABA, auxin, gibberellin, salicylic acid, and MeJA responsiveness identified; ABA-responsive elements occur in 90 of 94 genes (95.7%), excluding *SiLEA2-1*, *SiLEA2-33*, *SiLEA3-4*, and *SiASR-2*, underscoring ABA’s central role in regulating *SiLEA* expression.

### 3.5. Synteny Analysis of the Foxtail Millet SiLEA Genes

**Figure 5:**
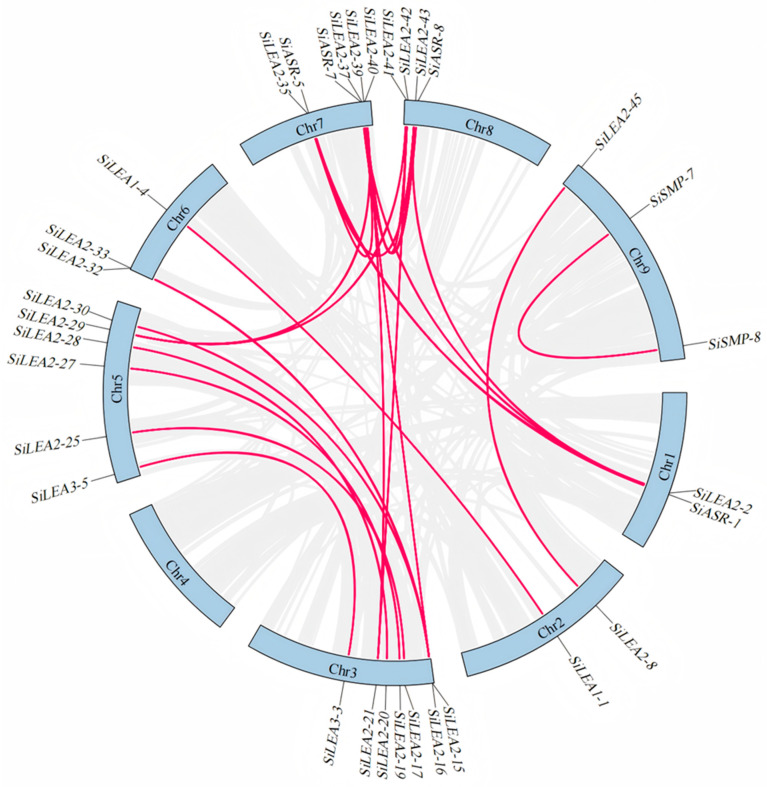
Synteny analysis of the *SiLEA* genes in foxtail millet.

To investigate the evolutionary mechanisms of the *SiLEA* gene family in foxtail millet, intra-species synteny analysis was performed (Figure 5). A total of 24 segmental duplicate gene pairs encompassing 33 *SiLEA* genes (35% of all family members) were identified (Table S4). Duplication patterns varied across subfamilies: the *SiLEA*_2 subfamily displayed a complex duplication network, featuring multi-gene duplication units (e.g., *SiLEA2-2* forming triplicate relationships with *SiLEA2-35*, *SiLEA2-37*, and *SiLEA2-43*) and bidirectional duplication pairs (e.g., *SiLEA2-15* with *SiLEA2-37* and *SiLEA2-33*; *SiLEA2-16* with *SiLEA2-30* and *SiLEA2-32*; *SiLEA2-21* with *SiLEA2-39* and *SiLEA2-42*; *SiLEA2-29* with *SiLEA2-40* and *SiLEA2-41*). *SiLEA2-37* served as a quadruplicate node, interacting with *SiLEA2-2*, *SiLEA2-15*, *SiLEA2-35*, and *SiLEA2-43*. Five large segmental duplication pairs were specific to the *SiLEA*_2 subfamily: *SiLEA2-8/SiLEA2-45*, *SiLEA2-19/SiLEA2-27*, *SiLEA2-20/SiLEA2-28*, *SiLEA2-17/SiLEA2-25*, and *SiLEA2-39/SiLEA2-42*. The *SiLEA*_1, *SiLEA*_3, *SiASR*, and *SiSMP* subfamilies contained segmental duplicates, including *SiLEA1-1/SiLEA1-4*, *SiLEA3-3/SiLEA3-5*, *SiASR-1/SiASR-5*, *SiASR-7*/*SiASR-8*, and *SiSMP-7/SiSMP-8*. Conversely, no duplication events occurred in the *SiLEA*_4, *SiLEA*_5, *SiLEA*_6, or *SiDehydrin* subfamilies. All duplicated gene pairs belonged to the same subfamilies, implying that chromosomal segment duplications likely drove their expansion, potentially resulting in functional redundancy.

Ka/Ks ratio analysis of segmental duplicate gene pairs in the *SiLEA* gene family revealed distinct evolutionary selection patterns (Table 2). Seventeen of the 24 homologous gene pairs with segmental duplication relationships (71%) exhibited Ka/Ks ratios significantly below 1 (*p* < 0.05), consistent with purifying selection. Six pairs (25%) displayed Ka/Ks ratios exceeding 1, potentially indicating positive selection. The *SiLEA2-8/SiLEA2-45* pair produced a Ka/Ks value of NaN due to synonymous site saturation, preventing accurate calculation of synonymous substitution rates. This implies these genes accumulated exceptional sequence variation, with coding regions divergence reaching the maximum measurable evolutionary distance.

**Figure 6:**
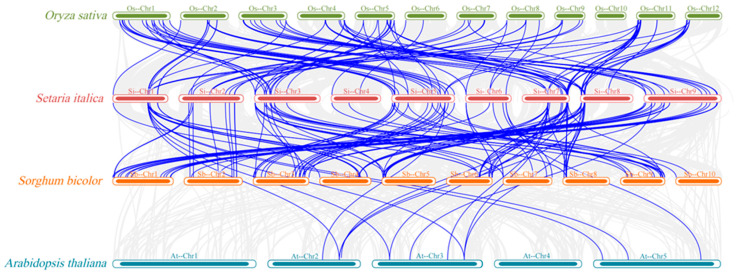
Synteny analysis of *LEA* genes in foxtail millet with *Arabidopsis*, rice, and sorghum.

To investigate the evolutionary dynamics of *LEA* genes in plant species and assess their divergence and conservation across taxa, cross-species synteny analysis was conducted using foxtail millet, sorghum (monocot C_4_ plants), rice (monocot C_3_ plant), and *Arabidopsis * (dicot plant). Foxtail millet shared significantly more orthologous *LEA* genes with sorghum than with rice, while both monocot comparisons showed substantially higher ortholog counts than the foxtail millet–*Arabidopsis* pairing, which yielded only 15 orthologous *LEA* genes (Figure 6). These results suggest that foxtail millet and sorghum diverged more recently, whereas foxtail millet and *Arabidopsis* exhibit greater phylogenetic distance in *LEA* gene evolution.

### 3.6. Tissue-Specific Expression Analysis of SiLEA Genes

**Figure 7:**
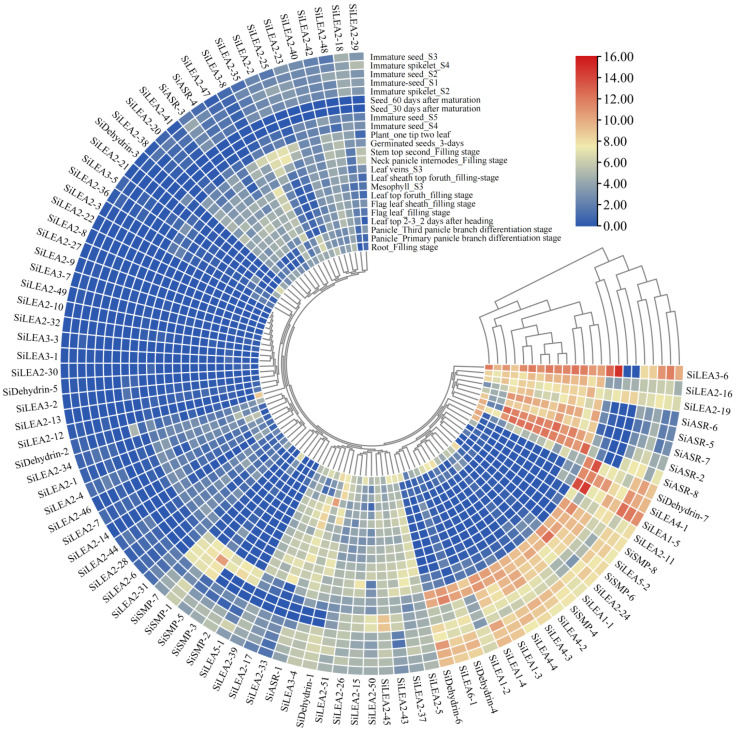
Tissue expression pattern analysis of *SiLEA* genes in foxtail millet.

The expression profiles of all 94 *SiLEA* genes across different tissues and developmental stages were analyzed using MDSi, revealing distinct spatiotemporal expression patterns (Figure 7, Table S5). *SiASR-8* was high expression in aerial tissues and during panicle development, while *SiSMP-5*, *SiLEA1-5*, and *SiLEA3-6* reached peak expression in immature seeds at stage S5 (Immature seed_S5). *SiLEA2-45* showed upregulation in mature seeds (30 and 60 days after maturation), whereas *SiLEA2-21* and *SiDehydrin-2* exhibited root-specific expression during the grain-filling stage. Elevated expression of *SiLEA3-4*, *SiASR-2*, *SiASR-5*, *SiASR-6*, and *SiASR-7* was observed in aerial tissues (Figure 7, Table S5).

**Figure 8:**
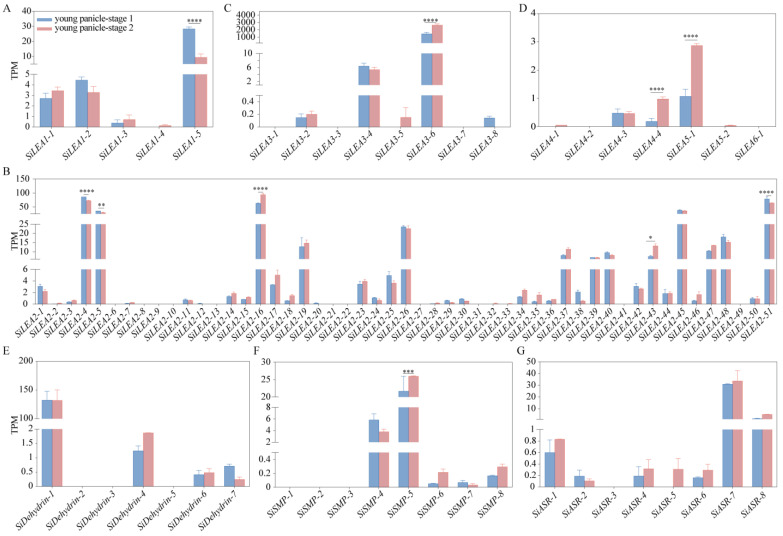
Expression patterns of *SiLEA*_1 (**A**), *SiLEA*_2 (**B**), *SiLEA*_3 (**C**), *SiLEA*_4—*SiLEA*_6 (**D**), *SiDehydrin* (**E**), *SiSMP* (**F**), and *SiASR* (**G**) subfamily genes in the panicles of foxtail millet. Young panicles of Jingu 21 were collected at two developmental stages: the branch meristem differentiation stage (young panicle stage 1, approximately 1.0–1.5 mm) and the distinct formation stage (young panicle stage 2, approximately 2.5–3.0 mm). Statistical significance was determined by *t*-test (*: *p* < 0.05, **: *p* < 0.01, ***: *p* < 0.001, ****: *p* < 0.0001). TPM: Transcripts Per Million.

Transcriptome analysis of foxtail millet panicles revealed distinct stage-specific expression patterns among *SiLEA* gene family members during two developmental phases (Figure 8). In the *LEA*_1 subfamily, *SiLEA1-5* transcripts were substantially more abundant than those of other members but decreased sharply during the young panicle-stage 2, while other genes remained stable (Figure 8A). The *LEA*_2 subfamily analysis showed reduced expression of *SiLEA2-4*, *SiLEA2-5*, and *SiLEA2-51* at young panicle-stage 2, contrasting with significant upregulation of *SiLEA2-16* and *SiLEA2-43* (Figure 8B). Among *LEA*_3–*LEA*_6/*PvLEA*18 members, *SiLEA3-6* maintained exceptionally high expression throughout both stages while other genes showed minimal activity; *SiLEA4-3* expression was stable, whereas *SiLEA4-4* and *SiLEA5-1* increased during stage 2 (Figure 8C,D). Within the *SiDehydrin*, *SiSMP*, and *SiASR* subfamilies, *SiDehydrin-1* expression remained stable, *SiSMP-5* exhibited coordinated upregulation, and *SiSMP-4* was significantly downregulated (Figure 8E–G). These results demonstrate the differential regulatory mechanisms operating across *SiLEA* subfamilies during panicle development.

ABA regulates root development in plants by activating transcriptional responses of specific genes. Distinct expression patterns emerged across *SiLEA* subfamilies following ABA treatment compared to controls. In the *LEA*_1 subfamily, *SiLEA1-2*, *SiLEA1-3*, and *SiLEA1-4* exhibited significant upregulation, with *SiLEA1-4* displaying the strongest induction (Figure S1A), suggesting its potential specialized role in ABA signaling. The *LEA*_2 subfamily exhibited ABA-induced upregulation in ten members—*SiLEA2-15*, *SiLEA2-16*, *SiLEA2-17*, *SiLEA2-19*, *SiLEA2-24*, *SiLEA2-25*, *SiLEA2-37*, *SiLEA2-45*, *SiLEA2-48*, and *SiLEA2-50*—while *SiLEA2-21* and *SiLEA2-35* were downregulated (Figure S1B). Five genes across the *LEA*_3–*LEA*_6/*PvLEA*18 subfamilies responded markedly to ABA: *SiLEA3-4* and *SiLEA3-6* (*LEA*_3), *SiLEA4-1* and *SiLEA4-3* (*LEA*_4), and *SiLEA5-2* (*LEA*_5) (Figure S1C,D). ABA treatment strongly induced *SiDehydrin-1*, *SiDehydrin-2*, *SiDehydrin-6*, and *SiDehydrin-7*—undetectable in untreated seedling roots—along with *SiSMP-4*, *SiSMP-5*, *SiASR-2*, *SiASR-7*, and *SiASR-8* (Figure S1E–G).

### 3.7. RT-qPCR Analysis of ABA-Responsive SiLEA Genes in Foxtail Millet

**Figure 9:**
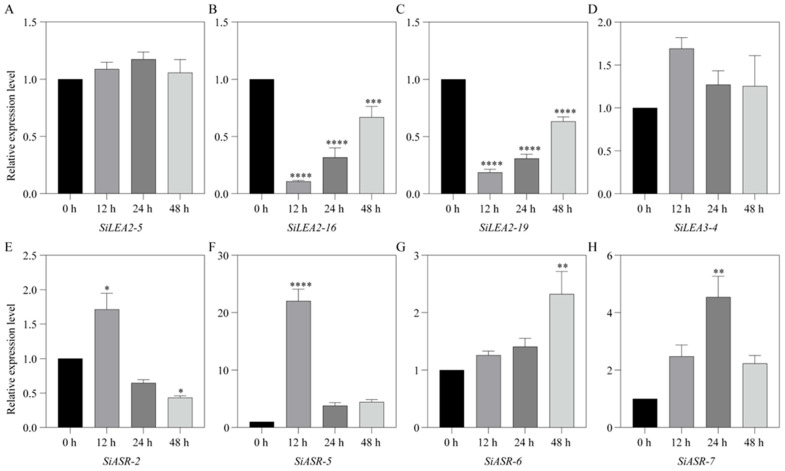
Expression patterns of *SiLEA2-5* (**A**), *SiLEA2-16* (**B**), *SiLEA2-19* (**C**), *SiLEA3-4* (**D**), *SiASR-2* (**E**), *SiASR-5* (**F**), *SiASR-6* (**G**), *and SiASR-7* (**H**) in the shoot of foxtail millet under ABA treatment. Three independent biological replicates yielded consistent results. Values represent means ± SE, with error bars showing replicate variability. Asterisks indicate statistically significant differences in *SiLEA* gene expression at 12 h, 24 h, and 48 h post-treatment relative to the 0 h untreated control (*t*-test; * *p* < 0.05, ** *p* < 0.01, *** *p* < 0.001, **** *p* < 0.0001).

Ten *SiLEA* genes—*SiLEA2-5*, *SiLEA2-16*, *SiLEA2-19*, *SiLEA2-21, SiLEA3-4, SiASR-2, SiASR-5, SiASR-6, SiASR-7*, and *SiDehydrin-2—*were selected for RT-qPCR validation to assess ABA responses in shoot and root tissues based on previous findings. Eight genes in shoots exhibited significant differential expression following ABA treatment (Figure 9). While *SiLEA2-5* remained stable, suggesting ABA-independent regulation (Figure 9A), *SiLEA2-16* and *SiLEA2-19* were initially downregulated at 12 h before gradually increasing (Figure 9B,C). *SiLEA3-4* exhibited a transient, though non-significant, upregulation (Figure 9D). The expression of *SiASR-2* and *SiASR-5* peaked at 12 h before declining (Figure 9E,F). In contrast, *SiASR-6* expression rose steadily, reaching maximum induction at 48 h (Figure 9G). *SiASR-7* peaked at 24 h (4.5-fold versus control) but partially declined to 2.2-fold by 48 h (Figure 9H).

**Figure 10:**
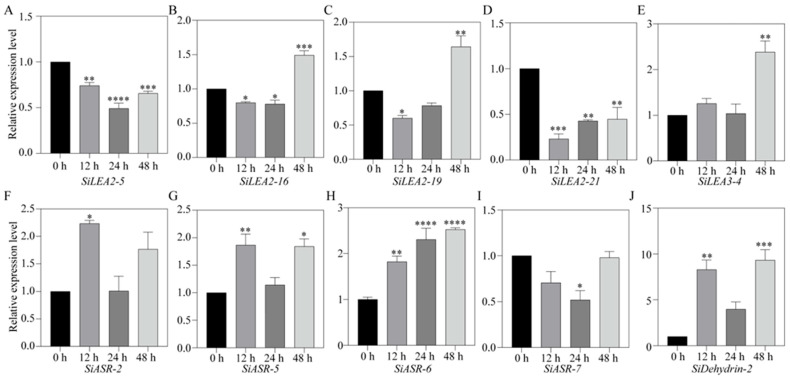
Expression patterns of *SiLEA2-5* (**A**), *SiLEA2-16* (**B**), *SiLEA2-19* (**C**), *SiLEA2-21* (**D**), *SiLEA3-4* (**E**), *SiASR-2* (**F**), *SiASR-5* (**G**), *SiASR-6* (**H**), *SiASR-7* (**I**), and *SiDehydrin-2* (**J**) in the root of foxtail millet under ABA treatment. All experiments were performed three times with consistent results. Data are presented as mean ± SE. The error bars indicate variation among replicates. Asterisks denote significant differences in the expression levels of these *SiLEA* genes at 12 h, 24 h, and 48 h after ABA treatment compared to the untreated 0 h control. Statistical significance was determined by *t*-test (*: *p* < 0.05, **: *p* < 0.01, ***: *p* < 0.001, ****: *p* < 0.0001).

All ten *SiLEA* genes were detected in roots under ABA treatment (Figure 10). *SiLEA2-5* expression declined under ABA treatment, reaching minimal levels by 24 h (Figure 10A). *SiLEA2-16* and *SiLEA2-19* expression decreased at 12 h but rebound to a 1.5-fold higher level by 48 h (Figure 10B,C). *SiLEA2-21* expression also decreased at 12 h but partially recovered thereafter (Figure 10D). *SiLEA3-4* activation occurred late, with expression peaking at 2.4-fold by 48 h (Figure 10E). *SiASR-2* expression was induced by ABA treatment at 12 h (Figure 10F). *SiASR-5* expression rose sharply at 12 h, returned to baseline at 24 h, and increased again by 48 h (Figure 10G). *SiASR-6* expression increased steadily, reaching its highest level at 48 h (Figure 10H). In contrast, *SiASR-7* expression decreased by 24 h (Figure 10I). *SiDehydrin-2* showed an expression pattern similar to *SiASR-5* (Figure 10J).

## 4. Discussion

The *LEA* gene family, which plays a crucial role in plant stress responses, has been well characterized in *Arabidopsis* [4], sorghum [5], rice [6], and wheat [7]. In this study, our bioinformatic analysis identified 94 *SiLEA* genes in foxtail millet, grouping them into nine subfamilies—*LEA*_1–*LEA*_6/P*vLEA*18, *ASR*, *Dehydrin*, and *SMP*— through comparisons of gene structure and conserved motifs. This classification mirrors earlier findings in *Arabidopsis* (nine subfamilies) [4], sorghum (eight subfamilies) [5], and rice (seven subfamilies) [6]. Notably, 53.19% of *SiLEA* genes contain no introns, exceeding the proportions observed in sorghum (47.05%) [5] and rice (35.2%) [6]. This structure feature could contribute to foxtail millet’s stress tolerance by enabling faster transcriptional responses, as intron-free genes bypass splicing delays during stress signal transduction [43]. Domain conservation patterns revealed subfamily-specific functions. SiLEA2-8 and SiLEA2-45 proteins contain the WHy domain, known for dehydration protection [44], whereas the *ASR* subfamily exclusively carries the ABA_WDS domain linked to ABA-mediated gene regulation [45,46].

Phylogenetic analysis placed *SiLEA* genes in close proximity to orthologs from monocot species such as sorghum and rice (Figure 2). The monocot-specific *LEA*_2 subfamily showed substantial expansion in foxtail millet (51 members), while the *Arabidopsis*-specific *AtM* subfamily (two members) was completely missing. This divergence probably arose from evolutionary adaptations in monocots, where gene duplication and functional specialization generated distinct stress-response mechanisms. Foxtail millet displayed specific enrichment of the *ASR* subfamily (eight members), which was absent in *Arabidopsis*. Since ASR proteins play essential roles in osmoregulation and ABA signal [45,46], their expansion in foxtail millet may enhance its drought tolerance. Synteny analysis demonstrated more orthologous *LEA* genes between foxtail millet and sorghum than between foxtail millet and either rice or *Arabidopsis* (Figure 6), indicating a more recent evolutionary divergence between foxtail millet and sorghum. This observation corresponds with the known genomic evolution of Poaceae species [5,6], implying that C_4_ plants likely faced comparable environmental pressures shaping *LEA* gene function.

Gene duplication drives the expansion and functional diversification of plant gene families [47]. In foxtail millet, segmental duplication events generated 35% of *SiLEA* genes (33 members), with the *SiLEA2* subfamily exhibiting an especially intricate replication network (Figure 5). *SiLEA2-37*, for instance, formed a quadruplicate replication node with multiple genes (e.g., *SiLEA2-2*, *SiLEA2-15*), reflecting frequent segmental duplication associated with polyploidization in Poaceae genomes [5]. Ka/Ks analysis revealed that 71% of duplicated gene pairs (e.g., *SiLEA2-16/SiLEA2-30*) evolved under purifying selection (Ka/Ks < 1), preserving their core functions. The remaining 25% of pairs (e.g., *SiLEA2-19/SiLEA2-27*) underwent positive selection (Ka/Ks > 1), suggesting potential functional innovations that may enhance stress adaptability in foxtail millet.

Integrated transcriptomic and RT-qPCR analyses revealed distinct tissue-specific and stress-inducible expression profiles of *SiLEA* genes in foxtail millet. *SiLEA2-21* and *SiDehydrin-2* showed elevated expression in roots (Figure 7), whereas *SiASR-5* and *SiASR-6* accumulated primarily in aerial tissues (Figure 7), suggesting functional specialization in stress adaptation. *SiLEA2-16*, *SiLEA2-19*, and *SiASR-6* exhibited pronounced ABA-induced upregulation in roots (Figure 10), with their promoter containing multiple ABA-responsive elements, implicating their role in ABA-dependent signaling. Conversely, *SiLEA2-5* and *SiLEA2-21* were significantly downregulated by ABA in roots (Figure 10). This functional divergence mirrors findings in rice (*OsEm1* and *OsLEA5*) [20,21], highlighting the coexistence of functional redundancy and regulatory specificity within the *LEA* gene family.

## 5. Conclusions

This study identified 94 *SiLEA* genes in foxtail millet through bioinformatic analysis, classifying them into nine subfamilies based on gene structures, conserved motifs, and phylogenetic relationships with *Arabidopsis*, rice, and sorghum. Sequences within each subfamily showed high conservation. Chromosomal mapping revealed uneven distribution of these genes across all nine foxtail millet chromosomes. Synteny analysis demonstrated segmental duplication events during *SiLEA* family evolution, while cross-species comparisons indicated closer orthologous relationships with the Poaceae species sorghum and rice than with the eudicot *Arabidopsis*. qRT-PCR analysis revealed that ABA regulates the expression of *SiASR-2*, *SiASR-5*, and *SiASR-6*, suggesting these genes in ABA-dependent signaling pathways. These results systematically characterize *SiLEA* genes’ features, expression profiles, and ABA responsiveness, establishing a framework for investigating their biological roles, decoding drought-resistance mechanisms, and developing stress-tolerant foxtail millet varieties.

## Figures and Tables

**Table 1:** Characterization of physicochemical features of *SiLEA* genes.

Gene Name	Gene ID	Genomic Location	Number of Amino Acids	Molecular Weight/KD	pI	GRAVY	Subcellular Localization
*SiLEA1-1*	*Si2g16530*	Chr2:23636372-23636878 (−)	168	16.54	9.39	−0.489	cyto
*SiLEA1-2*	*Si4g01700*	Chr4:588119-591858 (+)	125	13.49	9.84	−0.889	mito
*SiLEA1-3*	*Si4g12830*	Chr4:13541862-13542784 (−)	102	11.17	8.73	−1.012	nucl
*SiLEA1-4*	*Si6g11990*	Chr6:18263649-18264769 (+)	153	15.34	9.52	−0.722	nucl
*SiLEA1-5*	*Si7g22800*	Chr7:28695111-28695461 (+)	116	11.98	9.33	−0.928	mito
*SiLEA2-1*	*Si1g01120*	Chr1:336572-337207 (+)	211	21.66	10.67	0.08	golg
*SiLEA2-2*	*Si1g19260*	Chr1:26685069-26686200 (−)	212	22.88	9.23	0.212	E.R.
*SiLEA2-3*	*Si1g27150*	Chr1:34072139-34073281 (+)	194	21.25	9.81	0.128	mito
*SiLEA2-4*	*Si1g27250*	Chr1:34138008-34142334 (−)	351	37.84	10.19	−0.15	cyto
*SiLEA2-5*	*Si2g08600*	Chr2:7511879-7515924 (−)	254	27.19	9.16	0.209	chlo
*SiLEA2-6*	*Si2g10340*	Chr2:9405253-9405924 (−)	223	23.90	8.79	0.253	chlo
*SiLEA2-7*	*Si2g10360*	Chr2:9408452-9409066 (−)	204	21.63	9.75	0.116	cyto
*SiLEA2-8*	*Si2g11000*	Chr2:10739875-10741954 (−)	313	34.89	4.84	−0.39	cyto
*SiLEA2-9*	*Si2g16390*	Chr2:23233404-23234406 (+)	208	22.77	8.03	0.046	chlo
*SiLEA2-10*	*Si2g22270*	Chr2:31975586-31976874 (+)	262	28.69	10.12	0.093	chlo
*SiLEA2-11*	*Si2g28910*	Chr2:38432920-38434042 (−)	223	23.69	9.35	0.252	E.R.
*SiLEA2-12*	*Si2g33820*	Chr2:42245927-42247204 (−)	188	19.97	9.02	0.534	extr
*SiLEA2-13*	*Si2g33830*	Chr2:42252870-42253532 (−)	220	23.03	9.08	0.357	plas
*SiLEA2-14*	*Si2g34050*	Chr2:42443262-42444234 (+)	202	22.73	9.08	−0.071	chlo
*SiLEA2-15*	*Si3g01780*	Chr3:462543-463202 (+)	219	24.23	8.14	0.144	cyto
*SiLEA2-16*	*Si3g01790*	Chr3:466092-466709 (+)	205	22.00	9.03	0.286	chlo
*SiLEA2-17*	*Si3g11500*	Chr3:7657823-7660021 (−)	293	30.05	10.07	0.084	chlo
*SiLEA2-18*	*Si3g13070*	Chr3:8966951-8968514 (−)	240	24.84	10.26	0.225	cyto
*SiLEA2-19*	*Si3g13090*	Chr3:8974973-8975607 (+)	170	18.77	5.56	−0.155	nucl
*SiLEA2-20*	*Si3g16980*	Chr3:12605559-12606344 (+)	261	27.45	8.09	0.168	cyto
*SiLEA2-21*	*Si3g19790*	Chr3:15198175-15199340 (−)	214	22.66	10.33	0.43	plas
*SiLEA2-22*	*Si3g28330*	Chr3:27459991-27460596 (−)	201	20.54	9.41	0.514	chlo
*SiLEA2-23*	*Si5g01580*	Chr5:514279-515232 (−)	317	33.27	10.88	−0.295	chlo
*SiLEA2-24*	*Si5g02490*	Chr5:1883173-1883628 (+)	151	16.30	6.11	−0.028	cyto
*SiLEA2-25*	*Si5g14240*	Chr5:12401284-12403251 (−)	296	30.59	9.85	0.095	chlo
*SiLEA2-26*	*Si5g21720*	Chr5:27447618-27448559 (−)	313	32.30	11.03	−0.07	chlo
*SiLEA2-27*	*Si5g24710*	Chr5:30809909-30810829 (+)	175	19.06	5.01	−0.102	cyto
*SiLEA2-28*	*Si5g31440*	Chr5:36949347-36950099 (+)	250	26.85	5.30	0.006	cyto
*SiLEA2-29*	*Si5g35890*	Chr5:40487066-40488603 (+)	326	35.26	9.78	−0.12	chlo
*SiLEA2-30*	*Si5g39230*	Chr5:42968053-42969146 (−)	232	25.36	9.57	−0.14	E.R.
*SiLEA2-31*	*Si5g39250*	Chr5:42971994-42973145 (−)	241	26.70	9.49	−0.26	chlo
*SiLEA2-32*	*Si6g01320*	Chr6:331589-333238 (−)	274	29.90	8.55	0.37	plas
*SiLEA2-33*	*Si6g01330*	Chr6:334758-335402 (−)	214	23.46	9.23	0.30	cyto
*SiLEA2-34*	*Si6g19520*	Chr6:30784863-30786086 (−)	250	28.05	10.31	0.09	chlo
*SiLEA2-35*	*Si7g10930*	Chr7:19450879-19452721 (−)	219	23.63	8.20	0.28	extr
*SiLEA2-36*	*Si7g25560*	Chr7:30769688-30770332 (+)	214	23.72	9.68	−0.06	chlo
*SiLEA2-37*	*Si7g30030*	Chr7:34042880-34046298 (+)	209	22.95	8.28	0.137	chlo
*SiLEA2-38*	*Si7g30060*	Chr7:34053641-34054255 (+)	204	22.23	8.76	0.038	chlo
*SiLEA2-39*	*Si7g30680*	Chr7:34528844-34529524 (−)	226	24.16	9.43	0.123	chlo
*SiLEA2-40*	*Si7g31120*	Chr7:34820864-34836496 (−)	641	70.62	9.68	−0.299	E.R.
*SiLEA2-41*	*Si8g01520*	Chr8:523055-523933 (+)	292	31.66	10.99	−0.144	cyto
*SiLEA2-42*	*Si8g02080*	Chr8:876708-877391 (+)	227	24.16	9.43	0.176	chlo
*SiLEA2-43*	*Si8g04550*	Chr8:2883748-2884377 (−)	209	23.15	8.93	0.125	chlo
*SiLEA2-44*	*Si9g01150*	Chr9:272503-273129 (−)	208	22.08	9.12	0.084	chlo
*SiLEA2-45*	*Si9g02550*	Chr9:1053385-1056778 (+)	312	34.51	5.00	−0.372	cyto
*SiLEA2-46*	*Si9g02980*	Chr9:1300575-1301207 (−)	210	22.62	8.00	0.249	extr
*SiLEA2-47*	*Si9g02990*	Chr9:1322153-1322803 (−)	216	23.28	9.10	0.133	chlo
*SiLEA2-48*	*Si9g12880*	Chr9:8342829-8345960 (−)	266	29.27	9.30	−0.006	cyto
*SiLEA2-49*	*Si9g18470*	Chr9:13483147-13483770 (−)	207	22.10	8.97	0.263	cyto
*SiLEA2-50*	*Si9g20420*	Chr9:15461936-15464333 (+)	274	29.66	10.16	−0.111	E.R.
*SiLEA2-51*	*Si9g44540*	Chr9:50296499-50297353 (+)	236	24.85	9.23	0.358	chlo
*SiLEA3-1*	*Si1g20730*	Chr1:28206279-28206890 (+)	90	9.41	9.99	−0.186	chlo
*SiLEA3-2*	*Si3g20020*	Chr3:15406559-15407221 (−)	220	23.10	11.98	−0.709	nucl
*SiLEA3-3*	*Si3g26430*	Chr3:23674062-23679744 (+)	97	10.39	5.45	−0.218	chlo
*SiLEA3-4*	*Si5g03150*	Chr5:2342818-2343221 (+)	94	9.82	9.86	−0.339	chlo
*SiLEA3-5*	*Si5g03160*	Chr5:2347482-2347907 (+)	96	10.25	10.08	−0.165	chlo
*SiLEA3-6*	*Si5g06480*	Chr5:5284677-5284979 (+)	100	9.94	10.13	0	chlo
*SiLEA3-7*	*Si6g17070*	Chr6:28370327-28370969 (+)	96	10.28	9.18	−0.309	chlo
*SiLEA3-8*	*Si9g36850*	Chr9:44011390-44011988 (+)	133	14.63	9.62	−0.418	chlo/mito
*SiLEA4-1*	*Si3g16110*	Chr3:11735612-11736926 (−)	206	21.11	8.53	−1.176	nucl
*SiLEA4-2*	*Si7g24200*	Chr7:29717629-29719407 (+)	305	32.20	9.02	−0.89	chlo
*SiLEA4-3*	*Si9g41270*	Chr9:47738247-47740023 (+)	365	38.49	7.66	−0.936	mito
*SiLEA4-4*	*Si9g51210*	Chr9:55387138-55388377 (+)	370	38.08	7.73	−0.904	extr
*SiLEA5-1*	*Si3g26920*	Chr3:24540051-24540652 (−)	153	16.42	5.74	−1.380	nucl
*SiLEA5-2*	*Si3g26930*	Chr3:24544064-24545146 (−)	93	9.80	5.48	−1.280	nucl
*SiLEA6-1*	*Si2g25470*	Chr2:35395735-35396088 (−)	117	12.32	6.41	−0.925	nucl
*SiDehydrin-1*	*Si1g27340*	Chr1:34224000-34226135 (−)	499	54.80	9.14	−0.964	chlo
*SiDehydrin-2*	*Si5g17250*	Chr5:16894582-16895605 (−)	138	14.13	9.19	−1.178	nucl
*SiDehydrin-3*	*Si5g17260*	Chr5:16946844-16947365 (+)	138	14.10	8.90	−1.192	nucl
*SiDehydrin-4*	*Si5g29160*	Chr5:35231709-35232485 (+)	223	22.84	6.41	−0.843	nucl
*SiDehydrin-5*	*Si8g11920*	Chr8:21137495-21138260 (+)	186	19.21	9.68	−1.003	chlo
*SiDehydrin-6*	*Si8g11930*	Chr8:21187756-21189264 (−)	347	33.74	8.99	−0.804	nucl
*SiDehydrin-7*	*Si8g11970*	Chr8:21285230-21286503 (−)	169	16.91	8.81	−1.050	nucl
*SiSMP-1*	*Si1g21980*	Chr1:29302808-29303751 (−)	167	16.16	5.15	−0.139	cyto
*SiSMP-2*	*Si1g22010*	Chr1:29340219-29340650 (−)	143	14.12	5.88	−0.145	cyto
*SiSMP-3*	*Si1g22030*	Chr1:29358375-29358806 (−)	143	14.27	5.89	−0.176	cyto
*SiSMP-4*	*Si4g14860*	Chr4:21785714-21787597 (−)	288	28.98	4.63	−0.307	cyto
*SiSMP-5*	*Si6g13010*	Chr6:20828940-20830271 (+)	181	17.60	4.84	−0.317	cyto
*SiSMP-6*	*Si9g09570*	Chr9:5850754-5852099 (+)	342	34.68	6.00	−0.219	chlo
*SiSMP-7*	*Si9g24110*	Chr9:19527196-19528646 (−)	263	26.38	4.64	−0.419	nucl
*SiSMP-8*	*Si9g51720*	Chr9:55837685-55838706 (+)	280	27.75	4.65	−0.195	cyto
*SiASR-1*	*Si1g19510*	Chr1:27087304-27087745 (−)	107	11.82	6.76	−1.19	mito
*SiASR-2*	*Si5g45980*	Chr5:47661506-47662123 (+)	173	19.38	6.26	−1.277	mito
*SiASR-3*	*Si5g45990*	Chr5:47665510-47665827 (−)	105	11.71	9.73	−1.147	nucl
*SiASR-4*	*Si5g46000*	Chr5:47666703-47667492 (+)	102	11.54	9.80	−1.372	nucl
*SiASR-5*	*Si7g11260*	Chr7:19766534-19766938 (−)	101	11.48	6.81	−1.366	mito
*SiASR-6*	*Si7g11270*	Chr7:19769468-19771182 (−)	244	26.00	5.03	−1.636	nucl
*SiASR-7*	*Si7g29600*	Chr7:33748100-33749135 (+)	138	15.45	5.88	−1.30	mito
*SiASR-8*	*Si8g05130*	Chr8:3530617-3531156 (−)	137	15.42	6.15	−1.35	mito

**Table 2:** Ka/Ks ratios of segmental duplicated gene pairs in the SiLEA genes.

Homologous Gene	Non-Synonymous Substitution	Synonymous Substitution	Ka/Ks
*SiLEA2-2-SiLEA2-35*	0.25	0.57	0.44
*SiASR-1-SiASR-5*	0.1	0.46	0.21
*SiLEA2-2-SiLEA2-37*	0.4	0.34	1.18
*SiLEA2-2-SiLEA2-43*	0.42	0.35	1.19
*SiLEA1-1-SiLEA1-4*	0.38	0.68	0.56
*SiLEA2-8-SiLEA2-45*	0.19	NaN	NaN
*SiLEA2-19-SiLEA2-27*	0.24	0.42	0.56
*SiLEA2-20-SiLEA2-28*	0.3	0.4	0.75
*SiLEA2-17-SiLEA2-25*	0.28	0.41	0.68
*SiLEA3-3-SiLEA3-5*	0.49	0.64	0.76
*SiLEA2-16-SiLEA2-30*	0.88	0.65	1.34
*SiLEA2-16-SiLEA2-32*	0.44	0.43	1.03
*SiLEA2-15-SiLEA2-37*	0.45	0.44	1.02
*SiLEA2-15-SiLEA2-33*	0.31	0.53	0.58
*SiLEA2-21-SiLEA2-39*	0.55	0.6	0.92
*SiLEA2-21-SiLEA2-42*	0.55	0.63	0.88
*SiLEA2-29-SiLEA2-40*	0.61	0.62	0.98
*SiLEA2-29-SiLEA2-41*	0.51	0.48	1.06
*SiLEA2-35-SiLEA2-37*	0.39	0.49	0.79
*SiLEA2-35-SiLEA2-43*	0.48	0.53	0.91
*SiLEA2-39-SiLEA2-42*	0.02	0.08	0.24
*SiASR-7-SiASR-8*	0.11	0.24	0.47
*SiLEA2-37-SiLEA2-43*	0.18	0.43	0.42
*SiSMP-7-SiSMP-8*	0.34	0.51	0.67

## Data Availability

All data generated or analyzed during this study are included in this published article.

## Supplementary Materials

The following supporting information can be downloaded at: https://www.mdpi.com/article/10.3390/genes16080932/s1, Figure S1: Expression patterns of *SiLEA* genes in the root of foxtail millet; Table S1: Primers for RT-qPCR in this study; Table S2: *LEA* genes used for phylogenetic tree; Table S3: Major *cis*-regulatory elements in *SiLEA* gene promoters; Table S4: Tandem duplicated *SiLEA* genes; Table S5: The Transcripts Per Million (TPM) values of the *SiLEA* genes in different tissues.
