# Supplementary material for: Genome-Wide Identification and Expression Pattern Analysis of the Late Embryogenesis Abundant (LEA) Family in Foxtail Millet (Setaria italica L.)
Source: Genes (Basel). 2025 Aug 4;16(8):932. doi: 10.3390/genes16080932 (PMC12385335; doi:10.3390/genes16080932)
Supplement: Supplementary file 1 [file genes-16-00932-s001.zip › Figure S1, Table S1, Table S4.pdf]

Supplementary materials:

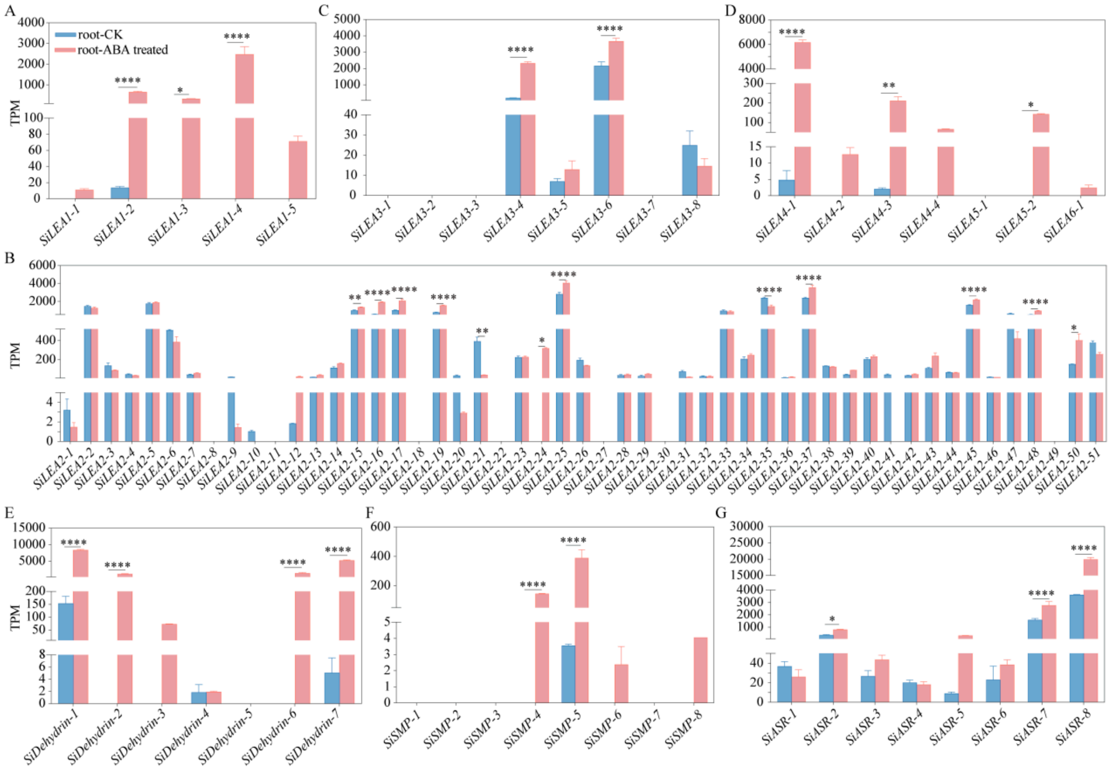

Figure S1. Expression patterns of *SiLEA* genes in the root of foxtail millet. Roots of 9-day-old Yugu1 seedlings (approximately 5 cm) treated with 2 μM ABA (root-ABA treated) and those without ABA treatment (root-CK) were collected. Statistical significance was determined by *t*-test (*: *P*<0.05, **: *P*<0.01, ****: *P*<0.0001). TPM: Transcripts Per Million.

Table S1. Primers for RT-qPCR in this study.

Gene name	Forward primer (5'-3')	Reverse primer (5'-3')
SiLEA2-5	GGC CAG GGA TGA GGT GTA TG	CAC AGG GAT GTG GAA ACG GA
SiLEA2-16	TTC AGG AAG GAG CAG ACC GA	GCT TGC AGG TGA TCT TAG CC
SiLEA2-19	GGA CTG GGA CAT CGA CTA CG	AGC ATG TCC TTG ATG GTC GG
SiLEA2-21	CTG GCG AGA TGG AGT TCG AG	TCC TGA CGC CGA ACT TTG AC
SiLEA3-4	TGC GTG AGC CTT CTA GCA C	GGT GTT CAC TTC CTT CTT GCC
SiASR-2	AGA GGC ACA AGA TCG AG	AGT TGC AGT AGT AGC CGT
SiASR-5	GCG AGG TCG ACT ACG AGA AGA	TGG CCT TGT GCT TCT CAT GC
SiASR-6	CGA AGA AGG ACC CGG AGA AC	TAG CCG AAG AAG TGG TGC TT
SiASR-7	CTC TTT ACG AGA AGC ACG AG	CCT TGT GGT CCT TCT TCT TC
SiDehydrin-2	AGG GCA TCA AGG AGA AGA TC	GGG AAG CTT CTC CTT GAT CT
Si9g37480	ATGGCCCGTACCAAGCAAACT	GGATCTCACGGAGGGCAACAGT

Table S4. Tandem duplicated *SiLEA* genes.

Gene Name	Gene ID	GeneName	Gene ID
SiLEA2-2	*Si1g19260*	*SiLEA2-35*	*Si7g10930*
SiASR-1	*Si1g19510*	*SiASR-5*	*Si7g11260*
SiLEA2-2	*Si1g19260*	*SiLEA2-37*	*Si7g30030*
SiLEA2-2	*Si1g19260*	*SiLEA2-43*	*Si8g04550*
SiLEA1-1	*Si2g16530*	*SiLEA1-4*	*Si6g11990*
SiLEA2-8	*Si2g11000*	*SiLEA2-45*	*Si9g02550*
SiLEA2-19	*Si3g13090*	*SiLEA2-27*	*Si5g24710*
SiLEA2-20	*Si3g16980*	*SiLEA2-28*	*Si5g31440*
SiLEA2-17	*Si3g11500*	*SiLEA2-25*	*Si5g14240*
SiLEA3-3	*Si3g26430*	*SiLEA3-5*	*Si5g03160*
SiLEA2-16	*Si3g01790*	*SiLEA2-30*	*Si5g39230*
SiLEA2-16	*Si3g01790*	*SiLEA2-32*	*Si6g01320*
SiLEA2-15	*Si3g01780*	*SiLEA2-37*	*Si7g30030*
SiLEA2-15	*Si3g01780*	*SiLEA2-33*	*Si6g01330*
SiLEA2-21	*Si3g19790*	*SiLEA2-39*	*Si7g30680*
SiLEA2-21	*Si3g19790*	*SiLEA2-42*	*Si8g02080*
SiLEA2-29	*Si5g35890*	*SiLEA2-40*	*Si7g31120*
SiLEA2-29	*Si5g35890*	*SiLEA2-41*	*Si8g01520*
SiLEA2-35	*Si7g10930*	*SiLEA2-37*	*Si7g30030*
SiLEA2-35	*Si7g10930*	*SiLEA2-43*	*Si8g04550*
SiLEA2-39	*Si7g30680*	*SiLEA2-42*	*Si8g02080*
SiASR-7	*Si7g29600*	*SiASR-8*	*Si8g05130*
SiLEA2-37	*Si7g30030*	*SiLEA2-43*	*Si8g04550*
SiSMP-7	*Si9g24110*	*SiSMP-8*	*Si9g51720*
